# Development of a standardized Gram stain procedure for bacteria and inflammatory cells using an automated staining instrument

**DOI:** 10.1002/mbo3.1099

**Published:** 2020-06-27

**Authors:** Hui Li, Lele Li, Yuanyuan Chi, Qingwu Tian, Tingting Zhou, Chunhua Han, Yuanqi Zhu, Yusun Zhou

**Affiliations:** ^1^ Department of Clinical Laboratory The Affiliated Hospital of Qingdao University Qingdao China; ^2^ Qingdao Women and Children's Hospital Qingdao China

**Keywords:** bacteria, Gram stain errors, inflammatory cells, molecular mechanism of Gram stain, standardized Gram stain procedure

## Abstract

Gram stain is a subjective and poorly controlled test, and the resultant errors often perplex laboratory scientists. To reduce errors and make Gram stain a precisely controllable and meritorious test, a standardized Gram stain procedure for bacteria and inflammatory cells was developed using an automated staining instrument in this study. Freshly expectorated sputum specimens, used as the optimized targets, were smeared on slides by laboratory technicians, defining each slide loaded with uniform matrix and monolayer cell. And then, the staining and decolorizing time, as well as the stain and decolorant volume, were optimized as 15, 105, 1, and 25 s and 1.1, 1.4, 0.3, and 0.7 ml, respectively. Culture‐positive blood specimens and original purulent fluids were used for confirming the developed standardized Gram stain procedure. Distinct tinctures of bacteria and inflammatory cells adhered to slide uniformly in a monolayer were observed, and the obtained staining results of these samples were highly consistent with their cultured results. Furthermore, according to the staining results under different staining conditions, an updated molecular mechanism of Gram stain for bacteria and the probable staining mechanism for inflammatory cells were also proposed in this study.

## Graphical abstract

In this study, a standardized Gram stain procedure for bacteria and inflammatory cells was developed using an automated staining instrument. Favorable staining results of clinical specimens highly consistent with their cultured results were obtained. Also, an updated molecular mechanism of Gram stain for bacteria and the probable staining mechanism for inflammatory cells were proposed.

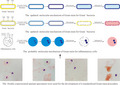

## 1 INTRODUCTION

Gram stain is one of the best‐known biological protocols to study microbes. Using this method, bacteria are easily distinguished from host cells in clinical specimens and differentiated into two classifications: gram‐positive (Gram^+^) and gram‐negative (Gram^−^). Gram stain can be executed immediately once the clinical specimen is smeared on a slide and briefly immobilized on a flame; so, it is simple, rapid, and cost‐saving. Compared with the popular molecular techniques, such as PCR, and the traditional culture methods, Gram stain can rapidly reveal almost all presumptive bacteria, even unusual or rare ones, and it is barely affected by antibiotic presence. Thus, it can be used to provide early diagnostic and therapeutic information for the control of bacterial infections (Boyanova, 2017). For instance, Gram stain has been applied to help with the rapid diagnosis of catheter‐associated bloodstream infections (Deleers et al., 2016), ventilator‐associated pneumonia (Gottesman et al., 2014), bacterial meningitis (Wu et al., 2013), urinary tract infection (Saadeh & Mattoo, 2011), and gonococcal urethritis (Taylor, DiCarlo, & Martin, 2011), with sensitivity higher than 69.6% and specificity greater than 77.8%. Furthermore, since different species of bacteria respond differently to some classes of antibiotics, Gram stain can help with the use of narrow‐spectrum antibiotics for initial empiric therapy, thus reducing the risk of the appearance of multidrug‐resistant bacteria (Taniguchi, Tsuha, Shiiki, & Narita, 2015).

The Gram stain, once classified as point‐of‐care testing, was performed and interpreted by clinicians in the past, but currently, the responsibility for the performance and interpretation of Gram stain has been delegated to general laboratory technicians, and clinicians have relied on microbiology laboratories for staining results. Without the assistance of important clinical information or the oversight from senior laboratory technicians, the skills of general technicians in the recognition of bacterial tincture and morphotype are deteriorating, which has increased Gram stain errors (Thomson, 2016). A relevant study that evaluated Gram stain errors in microbiology laboratories at four major tertiary hospitals revealed that the erroneous report rate was 0.4%–2.7%, and among the incorrectly interpreted specimens, respiratory specimen accounted for the highest proportion of 38% (Samuel, Balada‐Llasat, Harrington, & Cavagnolo, 2016). Another study assessing Gram stain performance and interpretation proficiency of general technicians at satellite laboratories found that the incorrect identification rate was 16% for bacteria in lower respiratory tract specimens (Munson, Block, Basile, Hryciuk, & Schell, 2007). Two other studies reviewing Gram stain errors showed that the error rate was 0.1%–1.3% for positive blood culture (Rand & Tillan, 2006) and 8.3% for cerebrospinal fluid (Tissot, Prod'hom, Manuel, & Greub, 2015).

Gram stain errors are attributed to a series of complex factors, including improper smear preparation, uncontrollable smear staining, and, consequently, incorrect smear interpretation (Samuel & Plebani, 2017). Laboratory scientists have taken steps to improve the Gram stain proficiency of laboratory technicians over the past few years. For example, Guarner et al. designed challenges that periodically required laboratory technicians to report tinctorial properties and morphological characteristics of different bacteria in several specimens provided by the reference microbiology laboratory, and the report errors were revised by laboratory scientists (Guarner, Street, Matlock, Cole, & Brierre, 2017). Owing to the frequent challenges, laboratory technicians' proficiency in Gram stain performance and interpretation could be improved. In contrast, Thomson and Siguenza et al. argued that, compared with repeated training for laboratory technicians to perform and interpret Gram stain, standardizing the staining procedure would be more efficient for reducing errors (Siguenza et al., 2019; Thomson, 2016). A proposed standard Gram stain procedure is that smearing specimen on a slide, defining the slide loaded with uniform matrix and monolayer cell, and then staining smears using an automated instrument under an optimized staining program (Baron, Mix, & Moradi, 2010). The two efforts could precisely control the staining time of Gram stain, effectively eliminate subjective disparity of manual staining, and finally provide uniform fields for smear interpretation.

In this study, using an automated staining instrument, a standardized Gram stain procedure for bacteria and inflammatory cells was developed, to take Gram stain from a subjective and poorly controlled test to a precisely controllable and meritorious test. Using the freshly expectorated sputum specimens as the stained objects, the influences of the staining and decolorizing time as well as the stain and decolorant volume on the tinctorial properties and morphological characteristics of bacteria and inflammatory cells were investigated thoroughly. Using the culture‐positive blood specimens and original purulent fluids as evaluated targets, the confirmation of the developed standardized Gram stain procedure was also conducted.

## 2 MATERIALS AND METHODS

### 2.1 Smear preparation

Freshly expectorated sputum specimens were acquired from patients with lower respiratory tract infection, and all the sputum samples used in this study were yellow, purulent, and not contaminated by the upper respiratory tract. The fresh sputum samples were stored at 4°C for less than 6 h before being transported to our microbiology laboratory. Laboratory technicians were required to repeatedly smear each sample on several slides until each slide was loaded with a uniform matrix and monolayer cell smear with its shape and area equivalent to that of a penny (diameter: ~19 mm). One sputum specimen was used for optimizing two variables (time and volume) of one reagent for Gram stain, with two slides for one of the chosen values in one variable, and another specimen was used for another reagent. For example, crystal violet is one reagent of Gram stain, and the same sputum specimen A was used for optimizing the staining time (7–68 s) and the stain volume (0.9–1.2 ml) of crystal violet. Sputum specimens B, C, and D were used for optimizing the variables of iodide solution, decolorant, and safranin O, respectively. When one variable was optimized, the others were kept constant.

### 2.2 Smear staining

The heat‐fixed smears were stained by an automated staining instrument (KS‐S100, Koreastandard Co., LTD.) that contained 20 horizontal cuvettes fixed on an alloyed disk and 5 pipelines for stains, decolorant, and deionized water. Each of the five pipelines corresponded to two symmetrical liquid outlets, which could realize simultaneous infusion for two cuvettes. The four reagents (Koreastandard Co., LTD.) included a solution of crystal violet in ethanol, a solution of iodine and iodide, a mixture of acetone and ethanol, and a solution of safranin O in ethanol. After slides were placed in cuvettes, an automated staining program was performed. The shape and size of the slide were fitted closely to that of the cuvette, and the entire slide could be moistened by a low volume of reagent that filled the cuvette in a specific order (crystal violet–iodide solution–decolorant–safranin O, with water washing after each step). The ejecting velocity for each reagent was a default value set by the instrument itself, and the number of slides placed in cuvettes could be adjusted from 1 to 20 using the *up* or *down* key on the operation panel. Staining was performed at room temperature, and after Gram stain, the instrument dried the stained slides by rotating the disk for 2 min. Laboratory technicians collected the dried slides and cleaned the cuvettes using 75% alcohol in deionized water (v/v).

### 2.3 Smear interpretation

After all the smears used for optimizing the automated staining program were stained, the senior laboratory technicians examined the stained smears with a Leica compound light microscope at low power (10 × 10) and then at oil‐immersed (10 × 100) field. The examiners observed the uniformity of smears and recorded the tinctorial properties and morphological characteristics of bacteria and inflammatory cells under different staining conditions (time and volume). Based on the examiners' descriptions, an optimized staining program was selected for the standardized Gram stain procedure. Under the optimal staining condition, distinct tinctures of bacteria and inflammatory cells in sharp contrast to ambient matrices were observed, and their original morphotypes were reserved integrally. Moreover, culture‐positive blood specimens and original purulent fluids were used for confirming the developed standardized Gram stain procedure.

## 3 RESULTS

### 3.1 Optimization of the automated staining program

#### 3.1.1 Staining and decolorizing time

The staining and decolorizing time that Koreastandard Co., Ltd. recommends is 70, 70, 5, and 30 s, respectively, for the four reagents. In this study, the chosen time intervals used for optimizing the staining and decolorizing time almost contained the recommended time values. The obtained staining results indicated that the time recommended by KS Corporation was not effective. Therefore, it is necessary to optimize the staining and decolorizing time for realizing distinct tinctures of bacteria and inflammatory cells (Table 1).

**TABLE 1:** The chosen staining and decolorizing time used for optimizing the automated staining program (the stain and decolorant volume was set as 0.9 ml recommended by KS Corporation; when one variable was optimized, the others were kept constant.)

Reagent		Variable: Staining and decolorizing time (s)					
		*Staining time of crystal violet*					
*Crystal violet*		*7*	***15***	*30*	*45*	*60*	*68*
	Iodide solution	90	90	90	90	90	90
	Decolorant	1	1	1	1	1	1
	Safranin O	25	25	25	25	25	25

		*Mordanting time of iodide solution*							
*Iodide solution*	Crystal violet	15	15	15	15	15	15	15	15
		*30*	*45*	*60*	*75*	*90*	***105***	*120*	*135*
	Decolorant	1	1	1	1	1	1	1	1
	Safranin O	25	25	25	25	25	25	25	25

		*Decolorizing time of decolorant*			
*Decolorant*	Crystal violet	15	15	15	15
	Iodide solution	105	105	105	105
		***1***	*2*	*3*	*4*
	Safranin O	25	25	25	25
		*Counterstaining time of safranin O*			
*Safranin O*	Crystal violet	15	15	15	15
	Iodide solution	105	105	105	105
	Decolorant	1	1	1	1
		*15*	***25***	*45*	*55*

**FIGURE 1:**
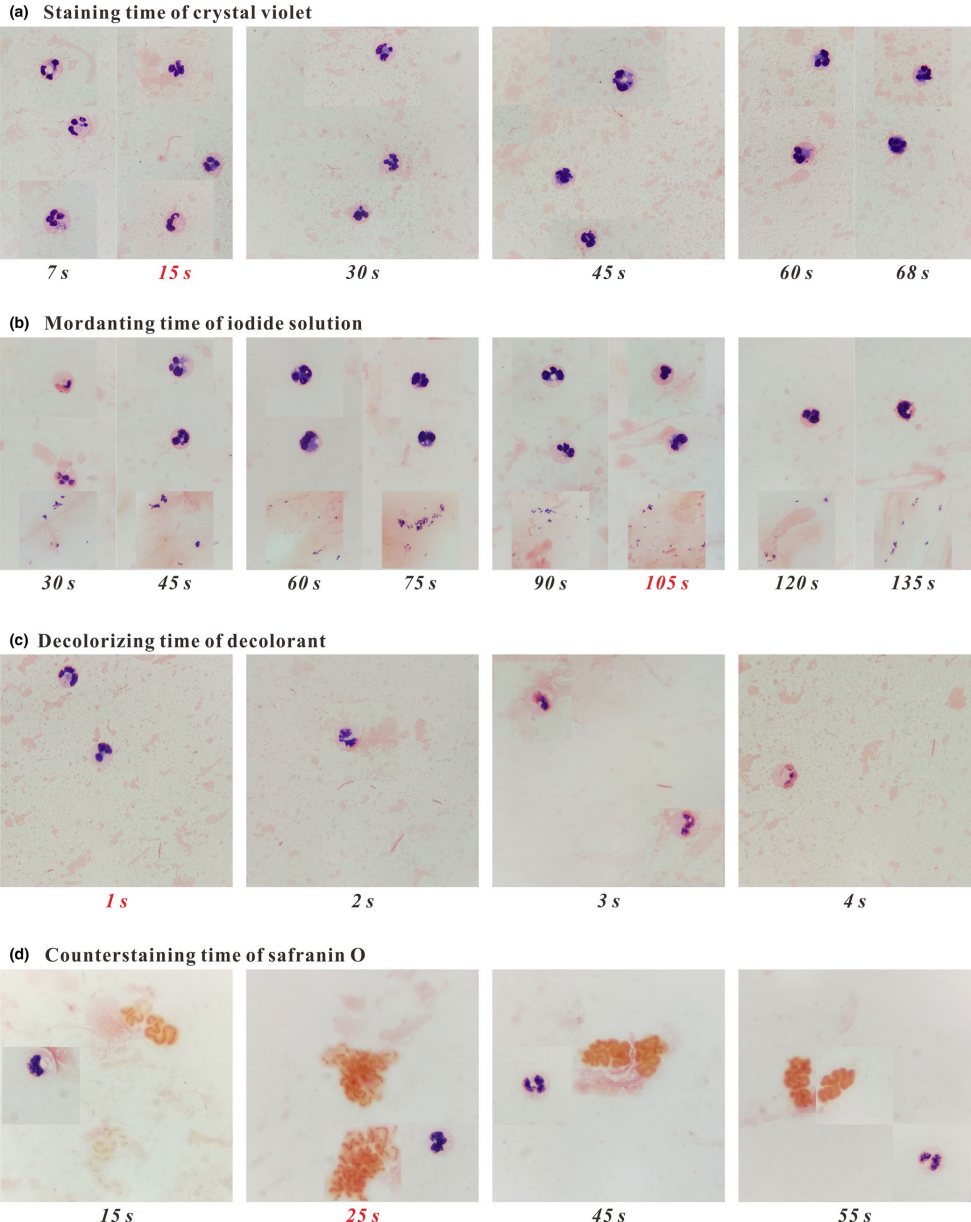
The influences of the staining and decolorizing time on the tinctorial properties and morphological characteristics of bacteria and inflammatory cells. The optimal times are red‐marked

The staining time (7–68 s) of crystal violet was first optimized. Figure 1a presents different tinctorial properties of inflammatory cells in specimen A from a patient who was diagnosed with a pulmonary infection caused by *Pseudomonas aeruginosa* (Gram^−^ bacilli) under several different staining times that had little influence on the tincture of Gram^−^ bacteria. When the staining time was set as 7, 15, or 30 s, the cell nuclei and cytoplasm of leukocytes were normally purple‐ and pink‐stained, respectively; when the staining time was 45, 60, or 68 s, the neutral granules were also purple‐stained and covered up the pink‐stained cytoplasm. Thus, the optimal staining time of crystal violet was selected as 15 s. Under this staining condition, bacteria, leukocytes, and ambient matrices were easily distinguished from each other.

Then, the mordanting time (30–135 s) of the iodine solution was optimized. Figure 1b shows the staining results of inflammatory cells and *Corynebacterium* spp. (Gram^+^ bacilli) in two specimens (B1 and B2). As shown in Figure 1b, in the range of time chosen for mordanting, Gram^+^ bacteria were normally purple‐stained, but leukocytes exhibited different tinctorial properties. When the smears were mordanted for 30 s, the cell nuclei were heterogeneously stained; when the smears were mordanted for 60 or 75 s, the cell nuclei and neutral granules were both purple‐stained, and the latter covered up the pink‐stained cytoplasm; and when the mordanting time was set as 45 or 90–135 s, the pink‐stained cytoplasm was clearly visible. Thus, the optimal mordanting time of the iodide solution was selected as 105 s for easily distinguishing bacteria, leukocytes, and ambient matrices.

Furthermore, the decolorizing time of decolorant was optimized. The tinctorial properties of inflammatory cells and *Klebsiella pneumonia* (Gram^−^ bacilli) in specimen C are presented in Figure 1c. With the increase of decolorizing time (1–4 s), the cell nuclei of leukocytes were stained as from purple to pink, while the morphotype of leukocytes was scarcely affected, and the pink‐stained property of Gram^−^ bacteria remained unchanged. Thus, the optimal decolorizing time was set as 1 s for easily differentiating bacteria, leukocytes, and ambient matrices. Under this decolorizing condition, the cell nuclei and cytoplasm of leukocytes were normally purple‐ and pink‐stained, respectively.

Finally, the counterstaining time of safranin O was optimized using two specimens (D1 and D2) from two patients both with bronchiectasis and respiratory tract infection caused by mucoid *P. aeruginosa* (Gram^−^ bacilli, with thick capsule). As shown in Figure 1d, the counterstaining time influenced the tinctures of inflammatory cells and Gram^−^ bacteria. With the increase of counterstaining time (15–55 s), Gram^−^ bacteria were stained as from light pink to dark orange until the dark‐stained capsule and thallus were hardly distinguished from each other; and for leukocytes, the tincture of cell nuclei turned from purple to pinkish‐purple, while the cytoplasm remained pink‐stained. Thus, the optimal counterstaining time was set as 25 s for easily differentiating the capsule and thallus of mucoid bacteria and making the cell nuclei of leukocytes normally purple‐stained.

#### 3.1.2 Stain and decolorant volume

The recommended stain and decolorant volume by KS Corporation is 0.9 ml for each reagent, with the attached advice that as long as the reagent completely moistens the slide, a satisfactory staining result could be obtained. However, they did not take into consideration the surface tension discrepancy of the four reagents and the ejecting velocity of the automated staining instrument for each reagent. Therefore, it is necessary to seek the appropriate stain and decolorant volume for obtaining satisfactory staining results (Table 2).

**TABLE 2:** The chosen stain and decolorant volume used for optimizing the automated staining program (the optimal staining and decolorizing time was used; when one variable was optimized, the others were kept constant.)

Reagent		Variable: Stain and decolorant volume (mL)			
		*Volume of crystal violet*			
*Crystal violet*		*0.9*	*1.0*	***1.1***	*1.2*
	Iodide solution	0.9	0.9	0.9	0.9
	Decolorant	0.9	0.9	0.9	0.9
	Safranin O	0.9	0.9	0.9	0.9

		*Volume of iodide solution*						
*Iodide solution*	Crystal violet	1.1	1.1	1.1	1.1	1.1	1.1	1.1
		*0.9*	*1.0*	*1.1*	*1.2*	*1.3*	***1.4***	*1.5*
	Decolorant	0.9	0.9	0.9	0.9	0.9	0.9	0.9
	Safranin O	0.9	0.9	0.9	0.9	0.9	0.9	0.9

		*Volume of decolorant*				
*Decolorant*	Crystal violet	1.1	1.1	1.1	1.1	1.1
	Iodide solution	1.4	1.4	1.4	1.4	1.4
		*0.9*	*0.7*	*0.5*	***0.3***	*0.1*
	Safranin O	0.9	0.9	0.9	0.9	0.9

		*Volume of safranin O*			
*Safranin O*	Crystal violet	1.1	1.1	1.1	1.1
	Iodide solution	1.4	1.4	1.4	1.4
	Decolorant	0.3	0.3	0.3	0.3
		*0.9*	*0.8*	***0.7***	*0.6*

Under the condition of the optimal staining and decolorizing time, the stain and decolorant volume was also optimized. We found that once the stain solution completely moistened the entire smear, increasing the stain volume did not influence the tinctures of bacteria and inflammatory cells. In this study, volumes of 1.1 ml, 1.4 ml, and 0.7 ml of crystal violet, iodide solution, and safranin O, respectively, could just completely moisten the slide. The volume difference was mainly ascribed to the surface tension discrepancy of the three stain solutions. Due to its low surface tension, only a volume of 0.2 ml of decolorant could moisten the entire slide, but no less than 0.3 ml of decolorant was needed to realize normal decolorization. In the practical application of the automated staining program, we put 20 smear‐loaded slides in each cuvette and found that with the increase of decolorant volume (0.5–0.9 ml), the number of over‐decolorized smears also increased. Thus, a volume of 0.3 ml of decolorant was taken as the optimal volume to realize normal decolorization for the 20 smears.

### 3.2 Sample storage temperature and time

Using the developed standardized Gram stain procedure, we investigated the influences of sample storage temperature (4 and 25°C) and time (6 and 12 h) on the tinctorial properties and morphological characteristics of bacteria and inflammatory cells in sputum specimens. We found that no matter how samples were stored, the tincture of bacteria was the same as that in fresh samples. As for inflammatory cells, only in sputum samples stored at 4°C for no more than 6 h could leukocytes have the same tincture and morphotype as those in fresh samples, and they tended to deform, loosen, and fracture, and became over‐decolorized as storage time went on. Thus, to obtain satisfactory staining results for both bacteria and leukocytes, samples should be stored at 4°C for less than 6 h.

### 3.3 Confirmation of the standardized Gram stain procedure

#### 3.3.1 Culture‐positive blood specimens

**FIGURE 2:**
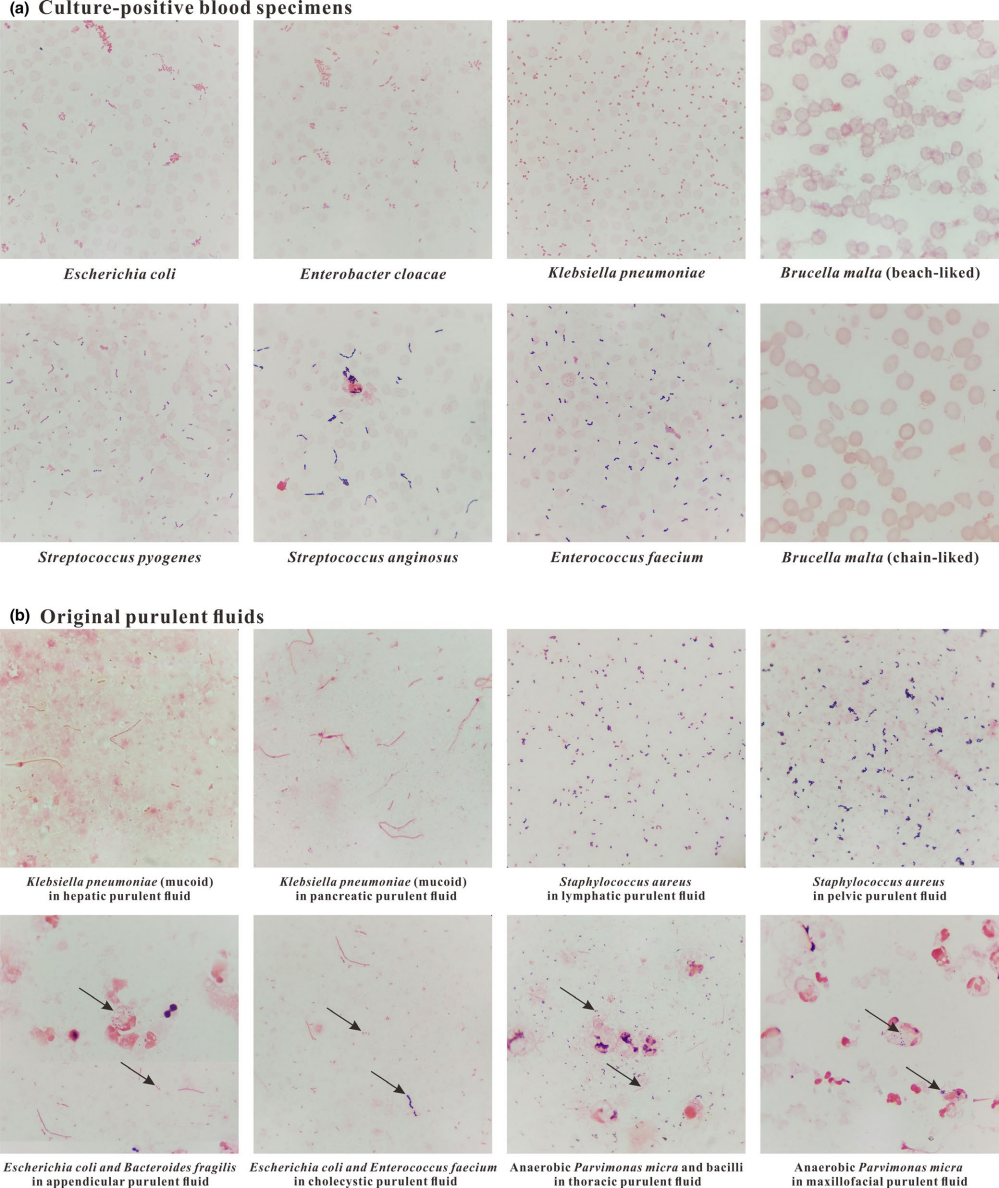
The staining results of the culture‐positive blood specimens and the original purulent fluids stained by the developed standardized Gram stain procedure

Culture‐positive blood specimens from bacteremic patients were stained by the standardized Gram stain procedure. Simultaneously, the positive blood samples were inoculated on Columbia blood‐agar and MacConkey‐agar plates. The obtained staining results of these samples were highly consistent with their cultured results. As presented in Figure 2a, bacteria, leukocytes, and erythrocytes adhered to the slide uniformly in a monolayer, and Gram^+^ and Gram^−^ bacteria were normally purple‐ and pink‐stained, respectively. Moreover, the phenomenon of hemolysis was easily found in blood samples infected with *Streptococcus pyogenes* (Gram^+^ cocci), and *Brucella malta* (Gram^−^ coccobacilli) arranging in a beach‐like or chain‐like form was also easily found in stained smears.

#### 3.3.2 Original purulent fluids

Original purulent fluids were also stained by the standardized Gram stain procedure. The purulent fluids were collected from the maxillofacial region, jugular lymphaden, thorax, hepar, cholecyst, pancreas, appendix, and pelvic cavity. The samples were inoculated on Columbia blood‐agar and MacConkey‐agar plates, and the obtained staining results were well matched up with their cultured results. As presented in Figure 2b, mucoid *K. pneumoniae* (with non‐colored capsule), swallowed *Bacteroides fragilis* (Gram^−^ anaerobic coccobacilli), and pathogenic *Parvimonas micra* (Gram^+^ anaerobic cocci) were easily found in stained smears. Also, different kinds of bacteria coexisting in a smear could be easily distinguished from each other.

## 4 DISCUSSION

The modern Gram stain procedure consists of four time‐sensitive steps: (a) staining heat‐fixed smears using positively charged crystal violet that electrostatically binds to available anionic substances; (b) introducing iodide solution as mordant to react with cationic crystal violet to generate stable precipitates; (c) removing the purple precipitates from Gram^−^ bacteria using decolorant but Gram^+^ bacteria still retain the precipitates by their thick peptidoglycan mesh (PM); and (d) counterstaining the decolorized smears using safranin O to make Gram^−^ bacteria pink‐stained and observing the counterstained smears with an optical microscope. As early as 1983, the molecular mechanism of Gram stain for bacteria has been explained by Beveridge and Davies (Beveridge & Davies, 1983). They proposed that both crystal violet and mordant could freely cross the outer membrane (OM) and cytoplasmic membrane (CM) of bacteria and the generated crystal violet‐mordant precipitates would accumulate within PM and cytoplasm. Until 2015, Wilhelm and coworkers provided new insight into the staining mechanism (Wilhelm et al., 2015). They have proved that in contrast to the conventional understanding that crystal violet could cross CM, this stain does not penetrate CM but only kinetically diffuses within PM. Therefore, the crystal violet‐mordant precipitates only accumulate in PM whose variations in Gram^+^ and Gram^−^ bacteria result in Gram‐variable responses.

**FIGURE 3:**
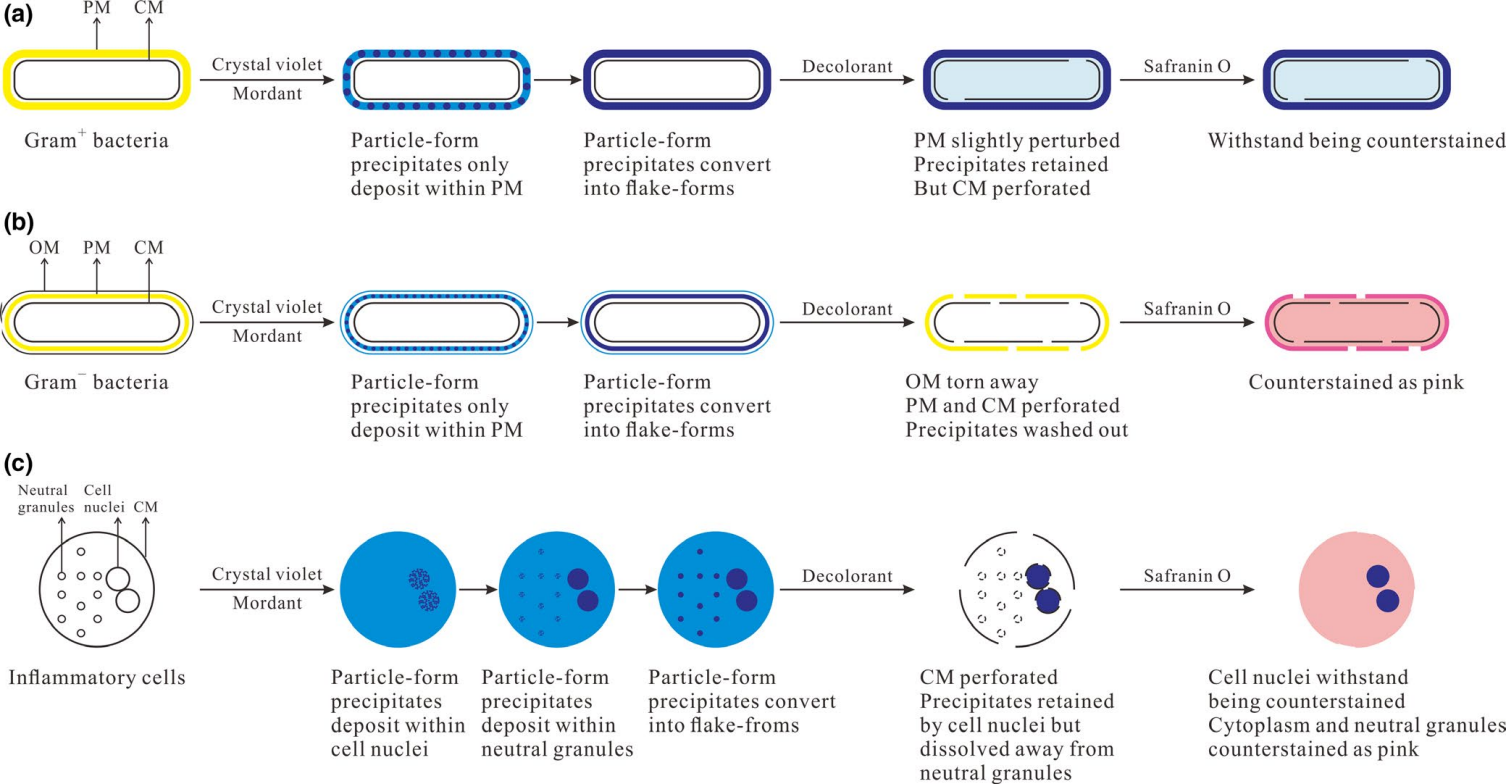
An updated molecular mechanism of Gram stain for bacteria and the probable staining mechanism for inflammatory cells

In this study, referring to the staining results of eukaryotes (inflammatory cells) under different staining conditions, we updated Wilhelm and coworkers' hypothesis by dividing the crystal violet‐mordant precipitates into particle‐ and flake‐form precipitates. The updated molecular mechanism of Gram stain for bacteria is presented in Figure 3a,b. (a) With the increase of mordanting time, the precipitates depositing in PM convert from particle‐ to flake‐form precipitates. The time spent on precipitates' form‐converting is influenced by the staining time of crystal violet, which embodies that the more time crystal violet stains bacteria, the more particle‐form precipitates will be generated in PM, and the more mordanting time should be required until all the particle‐form precipitates convert into flake forms. (b) At the decolorizing step, for Gram^−^ bacteria, OM is torn away from the cell, and CM and the thin PM are both perforated, which causes that the precipitates are easily dissolved away from the perforated PM. Conversely, for Gram^+^ bacteria, the thick PM is only slightly perturbed, so the precipitates are still retained in the cell wall by the intact PM and partially diffuse into cytoplasm across the small holes on the perforated CM. (c) At the counterstaining step, the retained precipitates help Gram^+^ bacteria withstand being counterstained by safranin O, while the decolorized Gram^−^ bacteria except for CM are counterstained as pink.

In this study, according to the staining results of inflammatory cells under different staining conditions, we generally deduced the probable molecular mechanism of Gram stain for inflammatory cells, which is presented in Figure 3c. (a) Crystal violet can freely penetrate the CM of leukocytes and binds to neutral granules by physical adhesion and negatively charged cell nuclei by electrostatic binding, resulting in accumulation of the crystal violet‐mordant precipitates within cell nuclei and neutral granules. (The more time of crystal violet staining leukocytes, the more precipitates will be generated in cell nuclei and neutral granules.) The time spent in precipitate depositing in PM, cell nuclei, and neutral granules is different, with quick deposition in PM, slow deposition in granules, and the deposition in nuclei somewhere in the middle. (b) With the increase of mordanting time, the precipitates deposit continuously and convert from particle‐ to flake‐form precipitates. We propose that sufficient mordanting time should be required until all the particle‐form precipitates convert into flake forms. Compared with particle‐form precipitates, the flake forms in neutral granules are easier to be dissolved away by decolorant. (If the mordanting time is not sufficient, some of the particle‐form precipitates will remain in neutral granules after decolorization.) Thanks to electrostatic binding instead of physical adhesion, the flake‐form precipitates in PM, and cell nuclei can resist being washed out by decolorant. (c) At the decolorizing step, the CM of leukocytes, neutral granules' membrane, and the cell nuclear membrane are all perforated by decolorant. The precipitates in neutral granules are dissolved away, while most of the precipitates in cell nuclei are retained, which is attributed to the electrostatic interaction between precipitates and cell nuclei and the big size of the cell nuclei beneficial for resisting being decolorized. (d) The retained precipitates in cell nuclei withstand being counterstained by safranin O, while the decolorized neutral granules are counterstained as pink. Ultimately, the cell nuclei of leukocytes are purple‐stained, and the cytoplasm and neutral granules are pink‐stained, which is beneficial to observe the endocytosis of inflammatory cells for bacteria.

Different from the staining results of leukocytes in fresh sputum specimens, deformed and over‐decolorized leukocytes were found in culture‐positive blood specimens. This was attributed to leukocytes tending to deform, loosen, and fracture during the sample culturing process and the cell nuclei in deformed leukocytes becoming loose and their ability to retain purple precipitates decaying. The over‐decolorized leukocytes were also found in original purulent fluids, which was mainly ascribed to leukocyte delivery to infected sites being slower than its apoptosis. Fortunately, bacteria in cultured and original clinical samples were normally stained because they could proliferate continually to generate new organisms. It is worth mentioning that although bacteria in clinical samples could tolerate long‐time storage, the PM of Gram^+^ bacteria would become thin and be easily heterogeneously stained once the growth phase of bacteria enters the decline period.

In conclusion, a standardized Gram stain procedure for bacteria and inflammatory cells was developed using an automated staining instrument in this study, and favorable staining results of clinical specimens well matched up with their cultured results were obtained. Also, an updated molecular mechanism of Gram stain for bacteria and the probable staining mechanism for inflammatory cells were proposed in this study. Thanks to the elimination of subjective influence on the staining process, the Gram stain errors that often perplex laboratory technicians will hopefully be reduced. Based on this standardized Gram stain procedure, a microbial image and character reporting system should be introduced, in which technicians send typical microscopic pictures and valuable descriptions to clinicians for rapid and accurate diagnosis of infectious diseases. We believe that these efforts may allow Gram stain to remain in the toolbox of every clinical microbiologist.

## CONFLICT OF INTEREST

None declared.

## AUTHOR CONTRIBUTIONS

**Hui Li:** Formal analysis (equal); investigation (equal); methodology (equal); writing – original draft (equal); writing – review & editing (equal). **Lele Li:** Formal analysis (equal); investigation (equal); visualization (equal); writing – original draft (equal); writing – review & editing (equal). **Yuanyuan Chi:** Investigation (equal); methodology (equal); resources (equal); visualization (equal). **Qingwu Tian:** Data curation (equal); project administration (equal). **Tingting Zhou:** Data curation (equal); project administration (equal). **Chunhua Han:** Investigation (equal); visualization (equal). **Yuanqi Zhu:** Conceptualization (equal); resources (equal). **Yusun Zhou:** Conceptualization (equal); funding acquisition (lead); supervision (lead); validation (lead); writing – original draft (equal); writing – review & editing (equal).

## ETHICS STATEMENT

This study was carried out following the guidance for the collection and testing of clinical samples. The protocol was permitted by the Medical Ethics Committee of the Affiliated Hospital of Qingdao University (Approval No.: QDFY20180512).

## Data Availability

All data generated or analyzed during this study are included in this published article.
